# Musculoskeletal Pain and Teleworking in Times of the COVID-19: Analysis of the Impact on the Workers at Two Spanish Universities

**DOI:** 10.3390/ijerph18010031

**Published:** 2020-12-23

**Authors:** Óscar Rodríguez-Nogueira, Raquel Leirós-Rodríguez, José Alberto Benítez-Andrades, María José Álvarez-Álvarez, Pilar Marqués-Sánchez, Arrate Pinto-Carral

**Affiliations:** 1 SALBIS Research Group, Department of Nursing and Physiotherapy, Campus de Ponferrada s/n, Universidad de León, 24400 Ponferrada, Spain; orodn@unileon.es (Ó.R.-N.); mjalva@unileon.es (M.J.Á.-Á.); mpmars@unileon.es (P.M.-S.); apinc@unileon.es (A.P.-C.); 2 Department of Nursing and Physiotherapy, Campus de Ponferrada s/n, Universidad de León, 24400 Ponferrada, Spain; 3 SALBIS Research Group, Department of Electric, Systems and Automatics Engineering, Campus of Vegazana s/n, Universidad de León, 24071 León, Spain; jbena@unileon.es

**Keywords:** pandemic, COVID-19, SARS-CoV-2, quality of life, physical activity, musculoskeletal pain, teleworking, public health, occupational risks

## Abstract

The special situation brought about by the coronavirus pandemic and the confinement imposed by the Government, has given rise to numerous changes in working habits. The workers at the universities have had to start a period of teleworking that could give rise to consequences for the musculoskeletal system. The objective of this article is to analyze the impact of the confinement on the musculoskeletal health of the staff of two Spanish universities. A cross-sectional, observational study was carried out on the workers. Data was taken in April–May 2020 and included: The Standardized Kuorinka Modified Nordic Questionnaire, the Perceived Stress Scale and another one on sociodemographic data. This study comprised 472 people. The areas of pain noted during the confinement period concluded that it was less in all cases (*p* < 0.001). The frequency of physical activity carried out increased significantly during the period of confinement (*p* < 0.04), especially in women. The type of physical activity done was also seen to modify during this period (*p* < 0.001), with a preference for strength training and stretching exercises. In conclusion, the confinement gave rise to changes in the lifestyle and in the musculoskeletal pain of the workers at the universities. All of this must be taken into account by health institutions and those responsible for the Prevention of Occupational Risks at Spanish universities.

## 1. Introduction

The special situation brought about by the coronavirus pandemic has given rise to numerous changes in living and working habits. The Spanish Government issued a decree on the 14 March 2020, adopting protection measures such as limiting the free movement of people, commercial activities not related to basic necessities, the cessation of face-to-face teaching and cultural, recreational, and sporting activities [1]. The workers at the universities have had to adapt themselves to the new situation of confinement with both the administrative staff (AS), and the teachers and research staff (TRS), who commenced a period of teleworking, due to the impossibility of maintaining close social contacts, necessary until then to carry out their work. Telework can be defined as work achieved with the help of Information and Communications Technology (smartphones, tablets, laptops, and desktop computers) and conducted outside the employer’s locations [2]. This new state has had a profound impact on the population both physically and psychologically [3].

Musculoskeletal disorders are a health problem related to office work (the most common type of work in Europe), affecting millions of workers [4]. In this sense, there is evidence that demonstrates that the ergonomic conditions in the workplace determine the musculoskeletal health of the workers [5,6], so it would be understandable that the change of location in which institutional work is carried out in a domestic setting could give rise to consequences for the musculoskeletal system. Among these consequences, previous studies have highlighted the conditions of the workspace with a greater prevalence of painful musculoskeletal disorders especially in the back [5,7].

Currently, the perception of pain is considered as the outcome of the interaction of physiological, emotional, cognitive, behavioral, and socio-cultural factors [8]. Pain is also the most frequent reason for medical consultation and at the time that it occurs it requires multidisciplinary attention [9]. Its impact is enormous since, for example, the pain caused by the dysfunction of the different spinal segments generates an annual average cost that amounts to more than one fifth of the total health expenditure of a country [10]. Furthermore, the relationship between anxiety, stress, and an inadequate style of coping with these difficult situations and the pain, has been established [11,12,13]. It is for this reason that the psycho-social factors, as well as the degree of physical activity (PA), are the two determinants with the greatest influence on the manifestation of musculoskeletal pain in workers [14,15,16]. In fact, Wang et al. [17] have quantified the psychological impact brought about by the outbreak of COVID-19, identifying that the prevalence of pathological degrees of stress has affected 28.8% of the population. Likewise, a study carried out in Spain during the confinement measures showed that 72% of the sample presented a psychological disorder which increased to 79% in women [18]. In the same study, the participants who had more physical symptomatology related to the presence of COVID-19, with headache, cough, myalgia, sore throat, or a cold, had a greater propensity to developing psychological disorders [18]. However, there are no records of studies that relate these data with a greater prevalence of musculoskeletal pain during the pandemic.

As regards the PA, a relationship has been established between less active lifestyle habits and musculoskeletal pain [19,20], which is why the prolonged confinement stage, during which the population were unable to leave their homes, thus prohibiting physical activity in specific areas for carrying it out, could lead to a change in physical activity habits and an increase in pain in the population. In the same way, sedentary behavior has been defined as any waking behavior characterized by an energy expenditure ≤ 1.5 METs, while in a sitting, reclining, or lying posture [21]. Screen time and sitting time are often the two main indicators used to quantify the time spent on sedentary behaviors. At the same time, sedentary activities that involve cognitive tasks (mental work) have the profile of an activity with very little movement and a neurogenic stress component [22,23].

At the same time, no studies have been found that determine the impact on musculoskeletal health derived from confinement and teleworking. For this reason, the carrying out of this study was considered necessary with the objectives of (a) analyzing the impact of teleworking during the confinement period on the musculoskeletal health of the workers of two Spanish universities; (b) determining whether the changes in type and frequency of PA that involved confinement changed the prevalence of musculoskeletal pain in the AS and TRS workers; and, finally, (c) to evaluate whether the said prevalence has a relationship with the changes in PA inherent to confinement and the stress self-perceived by the participants. With the initial hypotheses that teleworking during confinement worsened the musculoskeletal health of workers (increasing the prevalence of pain); and that the said increase in the prevalence of pain was related to the increase in sedentary behavior; and that the prevalence of musculoskeletal pain was higher among less active workers.

## 2. Materials and Methods

### 2.1. Study Design and Sample

A cross-sectional, observational study was carried out on the workers at two Spanish universities (the Universidad de León and the Universidad de Valladolid) with a convenience sample. The total population available in both universities was: 1535 AS workers (64.6% from the Universidad de Valladolid) and 3434 TRS workers (71.3% from the Universidad de Valladolid). Taking into account the total population, it was calculated that, to achieve a 99% level of confidence and a 10% margin of error, it was necessary to obtain the participation of 151 AS workers and 159 TRS workers [24]. The criteria for inclusion in the participation were (a) belonging to the body of workers at the Universities of León or Valladolid as AS or TRS; (b) the employment relationship with the University had a previous duration of at least six months; (c) remaining actively working from home (teleworking) during the confinement period due to the COVID-19 virus in Spain (that is, between 16.03.2020 and 11.05.2020); and (d) answering all of the questions included in the evaluation questionnaire and signing the informed consent form for participation in this study in accordance with the Helsinki Declaration (rev. 2013).

The gathering of data was carried out during the months of April and May 2020, by means of email, on the part of the respective Research Vice-Rectorates, with a digital link, through which the people who wanted to participate voluntarily were able to read and accept the informed consent form, as well as accessing the questionnaire created for this end. The data were treated in a totally anonymous way.

### 2.2. Variables in the Study

The collection of questionnaire data was used as an instrument which included:

(a) The Standardized Kuorinka Modified Nordic Questionnaire (SNQ) which was adapted to Spanish: an instrument to analyze the musculoskeletal symptoms in an ergonomic or occupational health context and measure the results of the epidemiological studies on musculoskeletal disorders [25,26] which has a good to very good test–retest reliability (k = 0.6–0.81), and internal consistency from good to acceptable (Kuder–Richardson 20 = 0.74–0.87), and a good construct validity [26]. The SNQ is divided into two parts, the general and the specific. In this study, only the general part was used which consists of 27 questions with Yes/No answers about any musculoskeletal symptoms during the last 12 months or the last seven days and about the impact on activities during the last 12 months. All of these questions refer to 9 areas: neck, shoulders, elbows, wrists/hands, the upper part of the back, the lower part of the back, hips/thighs, knees, and ankles/feet [25,27].

This part of the questionnaire is modified so that it complies with the circumstances and the context of this research. In this way the questions: “Have you had any problems in the last 12 months (discomfort, unease, or pain) in…?” was modified to, “Have you had any problems in the last 12 months prior to the confinement (discomfort, unease, or pain) in…?”; “During the last 12 months have you had any moment in which you were unable to carry out your normal work (from home or outside of it) as a result of the problem?” has been modified to, “During the last 12 months prior to the confinement have you had any moment in which you were unable to carry out your normal work (from home or you outside of it) as a result of the problem?”; the question, “Have you had a problem during the last 7 days?” has been modified to, “Have you had any problem (discomfort, unease, or pain) during the days that we were confined as a result of the coronavirus?” After the modification of the questionnaire a pilot test was carried out with a convenience sample of 15 people from the University community to check whether any aspect of the questionnaire needed to be reworded and if the questions were easily understood. The result of pilot test was that all of the participants had understood the questions and that was no need to reword the questions in it. The pilot test answers were not included in the results of this article.

(b) Perceived stress scale (PSS) in its version adapted to Spanish: Evaluates the degree to which the people perceive the demands of their environment as unpredictable and uncontrollable; that is, the perception of control over these demands. It had a reliability of (α = 0.82, test–retest, r = 0.77), and a suitable validity and sensitivity [28]. This questionnaire of 10 items is most frequently used in scientific literature to study the relationship between stress and psychological health [29,30].

(c) Frequency of doing PA: This aspect was polled by means of the questions, “Did you do PA before the confinement?” and “Did you do any PA during the confinement?” The options for answering were (1) never; (2) occasionally (some days a month); and (3) frequently (seven days a week).

(d) Type of PA done: This aspect was polled by means of the questions, “What type of PA did you mainly do before the confinement?” and “What type of PA did you mainly do during the confinement?” The options for answering were (1) nothing; (2) aerobic; (3) strength exercises; and (4) another type of exercise (such as stretching, for example).

(e) Other data of interest: sex, type of work (administrative or teaching and research); the average number of hours that you spend seated daily; the number of people with whom you lived in your home during the confinement; if you felt sadness or depression during the confinement; if you had musculoskeletal pain or not during the confinement, and if you needed to take medication or not as a result of musculoskeletal pain during the confinement.

### 2.3. Statistical Analysis

A descriptive analysis of all the study variables was carried out through the calculation of the average values (to determine the central tendency) and standard deviation (as a measure of dispersion).

The variables showed a normal distribution according to the Kolgomorov–Smirnov test (*p* > 0.05), and there was a homogeneity of variances, when applying the Levene test. The *t*-test was used to verify the existence of significant differences between the sexes and between the two groups of workers. The ANOVA test with the Bonferroni correction was done between the different groups of physical activity carried out divided by sex.

A correlation analysis was made between the PA carried out, the areas with pain manifesting and the perceived stress to find out the relationship between them.

We applied the logistic regression model (logit) to analyze the association of independent variables and the dependent dichotomous variable (musculoskeletal pain during the confinement: 0 = no; 1 = yes). Finally, the adjusted odds ratios (OR) with their confidence intervals were estimated using the multivariate regression model. The model was initially adjusted by age. The STATA program version 12 statistical package (StataCorp., College Station, TX, USA) was used for the statistical analysis and the statistical meaning was established at a value of *p* < 0.05 for all of the statistical tests.

## 3. Results

### 3.1. Descriptive Analysis and Comparison between Subgroups

This study included the data given by 472 people, of whom 283 were women (60%). There were 172 AS workers who were polled, (36.4%), and all the rest were TRS (Table 1). The response rate was 11.2% among AS workers and 8.7% among TRS workers. Between both sexes, significantly different characteristics were identified in the age, in the areas of pain perceived during the previous year, the limiting areas of pain for working activities during the previous year on the back and upper limbs, the areas of pain perceived during the confinement. As regards stress; in all the cases, it was the subgroups of women who produced the highest results (Table 1). On the other hand, the number of hours sitting down daily and the number of people with whom they live did not show significantly different results between the sexes.

The pre–posttest between the areas of pain during the previous 12 months and the areas of pain during the confinement obtained significant results for the sample as a whole and for the subgroups of workers and the sexes; it being less in all cases during the confinement (*p* < 0.001).

**Figure 1:**
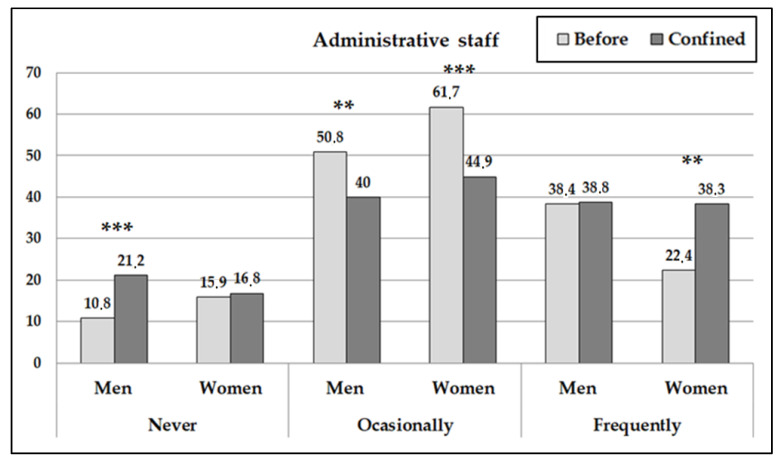
Percentages of administrative workers who carried out physical activity according to frequency before and after the confinement. (The asterisks indicate the significant differences before and during confinement by sex subgroup: ** *p* < 0.01; *** *p* < 0.001).

**Figure 2:**
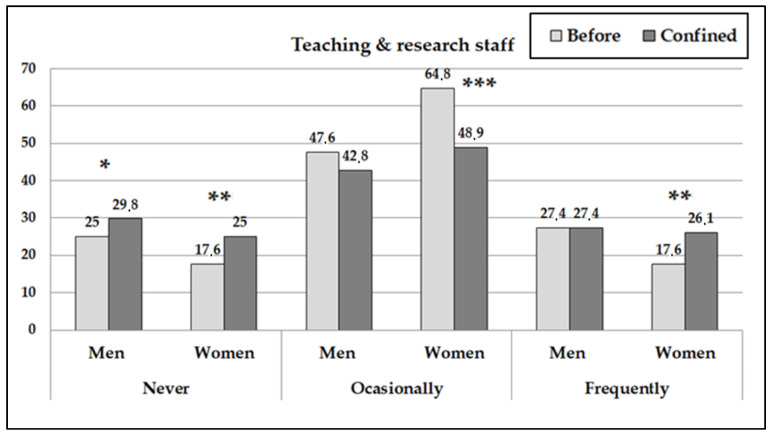
Percentages of teaching and research workers who carried out physical activity according to frequency before and after the confinement. (The asterisks indicate the significant differences before and during confinement by sex subgroup: * *p* < 0.05; ** *p* < 0.01; *** *p* < 0.001).

The frequency of PA carried out changed significantly during the period of confinement (contrast pre vs. posttest: *p* < 0.04) (Figure 1 and Figure 2). The TRS women increased more than 7% on not doing any exercise, at the same time those reducing the carrying out of occasional PA by 16 points and the number of women who carried out PA frequently increased by 9. On the other hand, the AS women showed an increase in the carrying out of frequent PA of 16%. In the analysis of men, they showed fewer changes in the carrying out of PA although an increase was identified in men who went from carrying out occasional exercise to not carrying out any exercise at all (more significant among the AS).

**Figure 3:**
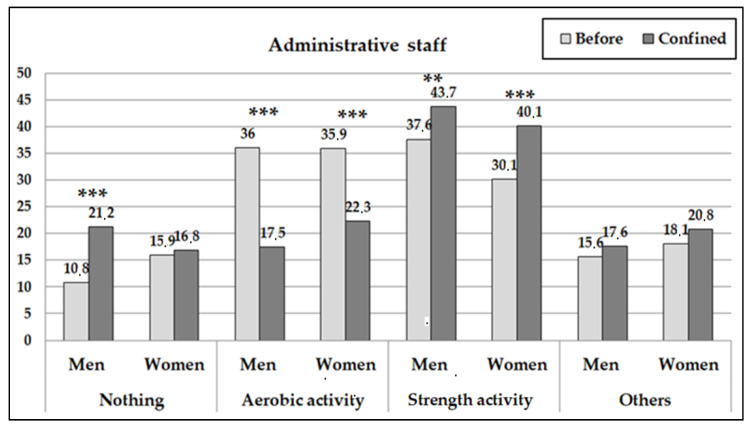
Percentages of administrative workers who carried out physical activity according to the type of physical activity carried out before and after the confinement. (The asterisks indicate the significant differences before and during confinement by sex subgroup: ** *p* < 0.01; *** *p* < 0.001).

**Figure 4:**
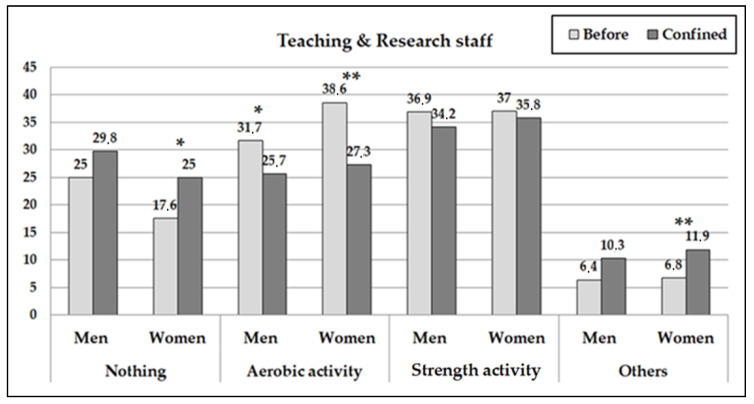
Percentages of teaching and research workers who carried out physical activity according to the type of physical activity carried out before and after the confinement. (The asterisks indicate the significant differences before and during confinement by sex subgroup: * *p* < 0.05; ** *p* < 0.01).

The type of PA done was also seen to modify during confinement (contrast pre vs. posttest: *p* < 0.001) (Figure 3 and Figure 4). The TRS women reduced the carrying out of aerobic PA significantly and increased the carrying out of other activities without reducing the proportion of women who trained with strength exercises. The AS women, on the other hand, significantly increased their training with strength exercises and other activities (replacing their aerobic training). The TRS men significantly reduced the carrying out of aerobic PA and strength exercises simultaneously on increasing doing no exercise at all and the carrying out of other activities. On the other hand, the AS men reduced the carrying out of aerobic exercises significantly and increased the carrying out of strength exercises and not doing any exercise at all.

The ANOVA analysis according to the type of PA carried out during the confinement was statistically significant for the age among the four groups of PA (*p* < 0.002, for the three comparisons) with the eldest doing other PA such as stretching (50 ± 10.3 years old), followed by those who carried out aerobic PA (48.5 ± 10.1 years old), those that carried out no PA at all (47.9 ± 10.7 years old) and those who carried out strength exercises (43 ± 11.6 years old).

The ANOVA analysis according to the type of PA carried out during confinement was also significant between the less active groups and the strength training groups according to the number of hours that they spent sitting down (the group doing strength exercises had an average of 6.7 hours sitting down daily and the least active group had an average of 7.4 hours). However, the type of PA carried out did not obtain significantly different results in any of the remaining variables used, the same as the frequency of PA (*p* > 0.05, in all of the cases).

### 3.2. Analysis of Correlation

The number of areas with pain during confinement correlated significantly with the number areas of pain during the previous 12 months for the total sample and for the subgroups of sex and position at work (0.6 < r > 0.7; *p* < 0.01 for all of them). On the other hand, the areas of pain during the confinement did not correlate with either the frequency of carrying out PA, or with the PSS (*p* > 0.05).

### 3.3. Analysis of Logistical Regression

The analysis of logistical regression indicated that the only variable with the capacity to influence the development of musculoskeletal pain was the sex (man = 0; woman = 1). Being a woman had a direct influence on the probability of developing this type of pain (OR = 2.363; *p* < 0.01). On the other hand, age, the frequency of PA during confinement, the amount of sitting down per day, the self-perceived stress and having feelings of sadness or anxiety were not significant in the regression model (Table 2).

## 4. Discussion

The objectives of this research were to analyze the impact of teleworking during the period of confinement on the musculoskeletal health of the workers of two Spanish universities; to determine whether the sedentary behavior that involved confinement changed the prevalence of musculoskeletal pain in the AS and TRS workers; and, finally, to evaluate whether the said prevalence has a relationship with the changes in PA inherent to confinement and the stress self-perceived by the participants. In the light of the results obtained, the prevalence of musculoskeletal pain among the University workers studied appears to have reduced during the time in which they were confined and carrying out teleworking. At the same time, it seems that they modified their PA habits as regards frequency and the type of activities carried out. On the other hand, these variables do not appear to be associated, in any case, with the self-perceived level of stress.

Specifically, in the areas with musculoskeletal pain, these were more numerous in women, both in the 12 months prior to the confinement and during it (although they decreased during the confinement in all of the subgroups). This higher prevalence in areas of pain in women is a finding which is in agreement with other studies which indicate that women who work with computers suffer more musculoskeletal pain [5,31].

As regards the modification of the PA habits, the most relevant finding is that the number of women who carry out exercise frequently has increased. The significant increase in women who went from carrying out occasional exercise to frequent exercise could help in the reduction in the areas of pain during the confinement [32]. PA is one of the most efficient methods of treatment for musculoskeletal pain [33]. On the other hand, one of the most important predisposing factors for musculoskeletal pain is sedentary behavior [34]. Guidelines recommend a minimum of 30 min of moderate activity, five days a week, or 20 min of vigorous physical activity three days a week, so the increased frequency of PA could explain the reduced prevalence of musculoskeletal pain [35,36]. This change in PA habits, especially in the case of women, may give rise to a greater control of conciliation between working hours and domestic duties thanks to teleworking; particularly in those women who combine their work tasks with the carrying out of housework and looking after their children [37,38].

In fact, the results obtained through the analysis of the logistical regression corroborate the differences detected in the development of musculoskeletal pain in both sexes. Studies have already confirmed that health in men and women is different due to biological factors (genetics, hereditary, and physiological) and social [39,40]. These differences are reflected in the “paradox of morbidity”, which is why men die younger, but women live longer with worse health [41,42].

The greatest changes in the carrying out of PA come about in relation to the decline in doing aerobic activity. The cause of this phenomenon would be that aerobic exercise is usually carried out in the open air (running, cycling, walking, etc.) [43]. Consequently, the majority of those questioned in this research substituted aerobic exercise with strength exercises or others, such as stretching and yoga. Previous research has indicated that the greatest benefits as regards the treatment and prevention of musculoskeletal pain are obtained by doing strength or stamina exercises, coordination and stabilization [44]. Therefore the change in the type and frequency of PA might justify the improvement in the painful symptomatology of the population in the study. On the other hand, there is consensus that exercise should be individualized and based on the preferences of the person and, which should be perceived as safe rather than threatening in order to avoid the formation of incorrect associations between the carrying out of PA and pain [45].

The subject of future research could be to research if the change in the PA habits for a routine that was chosen and accepted by the workers (generating a sensation of control and self-accuracy), carried out in the most convenient timetable which included strength exercises and stretching; as well as having carried it out in the most frequent way, probably reaching the parameters recommended by the World Health Organization, may have improved the areas of pain in the population of the University workers studied.

This study has significant limitations which must be recognized. First, self-reported information on stress and painful areas has been used instead of measurements taken by an expert evaluator through objective and validated instruments. Secondly, the information about the frequency and type of PA carried out has also been self-reported rather than using objective information facilitated by accelerometers or pedometers (to avoid the risk of social desirability in the answers provided). It would also have been very useful to have an estimation of the stress prior to the confinement (which has been established by the number of painful areas reported). Furthermore, the authors acknowledge that the methodology used limits the generalization of the results (invalid outcome measures by changing the SNQ, no coincidences can be assessed by cross-sectional studies, nor can blinding or the recall bias). Those limitations respond to the exceptional situation of confinement in which the researchers also find themselves. Finally, as the population in the study belongs exclusively to two Spanish universities which limits the generation of our results to other populations.

In spite of the aforementioned limitations, there are also significant strengths. It is a wide-ranging population study, although with caution, it is representative of the population of Spanish University workers and a real reflection on the situation lived during the confinement in Spain. At the same time, this is the first time that direct relationships have been detected between the confinement, the PA habits of the workers and prevalence of musculoskeletal pain, giving rise to variables and associations that behave in a similar way to other situations but, in other cases, giving rise to particularities in the population of the study: it seems that there was an increase in the frequency of carrying out PA and a change towards strength training and stretching exercises (although these findings are subject to implicit bias in the measurement instruments used).

## 5. Conclusions

The confinement imposed in Spain between March and May 2020 gave rise to changes in the lifestyle of the population. Specifically, between the teachers, researchers, and administrative staff of universities there was an increase in the frequency of carrying out PA (especially women) and a change between the preference for aerobic activities prior to the confinement towards strength training and stretching exercises during this period. At the same time, a reduction has been identified in the prevalence of musculoskeletal pain.

All of this must be taken into account by health institutions and those responsible for the Prevention of Occupational Risks at Spanish universities in the face of implementing measures that encourage the carrying out of PA (quantity and suitable ways) which comply with the health regulations for the promotion of good health, improve the quality-of-life of the workers and reduce the cost related to work incapacity brought about by musculoskeletal pain. All of this always presents the physiological and socio-family differences between both sexes.

## Figures and Tables

**Table 1:** Descriptive statistics of the sample (data provided: mean ± standard deviation).

	All (*n* = 472)		Administrative Staff (*n* = 172)		Teaching and Research Staff (*n* = 300)	
	Men (*n* = 189)	Women (*n* = 283)	Men (*n* = 65)	Women (*n* = 107)	Men (*n* = 124)	Women (*n* = 176)
Age (years)	48.1 ± 10.9 **	45.3 ± 11.2 **	48 ± 10.6	47.7 ± 11.2	48.1 ± 11.1 **	43.9 ± 11 **
Daily sitting time (hours)	7 ± 2.5	6.9 ± 2.3	6 ± 2.6	6 ± 2.3	7.6 ± 2.3	7.4 ± 2.1
People with whom they live (*n*)	2.1 ± 1.4	2 ± 1.2	2.3 ± 1.2	2 ± 1.1	2 ± 1.6	1.9 ± 1.2
PA12 (*n*)	2.9 ± 1.9 **	3.5 ± 2.4 **	3.1 ± 2	3.6 ± 2.5	2.8 ± 1.9 **	3.5 ± 2.3 **
PA12-S (*n*)	1.3 ± 1 **	1.5 ± 1.1 **	1.4 ± 0.9 *	1.6 ± 1.1 *	1.3 ± 0.9	1.6 ± 1
PA12-LL (*n*)	0.8 ± 0.6	0.9 ± 0.6	0.9 ± 0.7	0.9 ± 0.8	0.8 ± 0.6	0.8 ± 0.7
PA12-UL (*n*)	0.8 ± 0.6	0.8 ± 0.8	1.1 ± 1.1	1.3 ± 1.2	1.1 ± 0.9	1.3 ± 1.3
AL12 (*n*)	1 ± 0.5 *	1.3 ± 0.7 *	0.8 ± 0.5 **	1.5 ± 0.9 **	1.1 ± 0.5	1.2 ± 0.6
AL12-S (*n*)	0.6 ± 0.3 ***	0.7 ± 0.4 ***	0.5 ± 0.2 *	0.8 ± 0.5 *	0.6 ± 0.3	0.6 ± 0.3
AL12-LL (*n*)	0.4 ± 0.1	0.4 ± 0.1	0.4 ± 0.1	0.5 ± 0.1	0.4 ± 0.1	0.4 ± 0.1
AL12-UL (*n*)	0.4 ± 0.1 *	0.5 ± 0.2 *	0.2 ± 0.2 **	0.5 ± 0.2 **	0.3 ± 0.1 *	0.5 ± 0.2 *
PAC (*n*)	2.5 ± 1.7 **	3 ± 2.1 **	2.5 ± 1.7	2.8 ± 2.3	2.4 ± 1.7 **	3.1 ± 2 **
PAC-S (*n*)	1.3 ± 0.9 *	1.6 ± 1 *	1.3 ± 1	1.4 ± 1.1	1.3 ±1 *	1.6 ± 1 *
PAC-LL (*n*)	0.8 ± 0.6	0.9 ± 0.7	0.8 ± 0.6	0.9 ± 0.7	0.8 ± 0.5	0.6 ± 0.6
PAC-UL (*n*)	0.8 ± 1	1.2 ± 1	0.7 ± 0.7	0.8 ± 0.8	0.8 ± 0.6	0.8 ± 0.8
PSS (score)	19.2 ± 4.8 **	20.6 ± 4.9 **	17.5 ± 5.3 *	19.4 ± 5.1 *	20 ± 4.3 *	21.4 ± 4.7 *

PA12: painful areas during the previous 12 months; PA12-S: painful area in the spine during the previous 12 months; PA12-LL: painful areas in the lower limbs during the previous 12 months; PA12-UL: painful areas in the upper limbs during the previous 12 months; AL12: areas with limiting pain during the previous 12 months; AL12-S: areas with limiting pain in the spine during the previous 12 months; AL12-LL: areas with limiting pain in the lower limbs during the previous 12 months; AL12-UL: areas with limiting pain in the upper limbs during the previous 12 months; PAC: painful areas during confinement; PAC-S: painful areas in the spine during confinement; PAC-LL: painful areas in the lower limbs during confinement; PAC-UL: painful areas in the upper limbs during confinement; PSS: Perceived stress scale. *t*-test between sex: * *p* < 0.05; ** *p* < 0.01; *** *p* < 0.001.

**Table 2:** Binomial Logistic Regression of musculoskeletal pain in relation to gender, physical activity practice and emotional perception.

Variable	OR	SE	*p*	95% CI
Sex	2.363	0.761	0.008	1.258–4.441
Age	0.991	0.013	0.467	0.966–1.016
Frequency of physical activity during confinement	0.914	0.173	0.63	0.63–1.33
Perception sadness or anxiety	0.989	0.012	0.36	0.96–1.01
Self-perceived stress	1.017	0.03	0.57	0.96–1.08
Amount of sitting down per day	0.934	0.065	0.09	0.86–1.01

OR: odds ratio; SE: standard error; 95% CI: 95% confidence interval.
